# Lessons from Nigeria’s Adaptation of Global Health Initiatives during the COVID-19 Pandemic

**DOI:** 10.3201/eid2813.221175

**Published:** 2022-12

**Authors:** Chikwe Ihekweazu

**Affiliations:** World Health Organization, Berlin, Germany

**Keywords:** COVID-19, respiratory infections, severe acute respiratory syndrome coronavirus 2, SARS-CoV-2, SARS, coronavirus disease, zoonoses, viruses, coronavirus, global health, public health, disease outbreaks

## Abstract

Nigeria receives funds from several global health initiatives that are aimed at addressing elevated risks and overall burden of infectious disease outbreaks. These funds include the Global Fund to Fight AIDS, Tuberculosis and Malaria; US President’s Emergency Plan for AIDS Relief; US President’s Malaria Initiative; and Global Polio Eradication Initiative. These initiatives have contributed to a substantial reduction in illness and death from HIV, tuberculosis, malaria, and polio. However, Nigeria has experienced mixed success with leveraging the capacities built through these donor-funded vertical programs to respond to new health threats. This report describes experiences using resources from vertical disease programs by the Nigeria Centre for Disease Control in response to the 2014–2016 Ebola outbreak in West Africa and the COVID-19 pandemic. Integrating resources from different disease programs with government-led systems and institutions will improve responses to endemic outbreaks and preparedness for future pandemics in Nigeria.

Similar to other countries in Africa, Nigeria receives substantial donor funds through global health initiatives aimed at addressing the high prevalence of infectious diseases and other public health threats (*1*). These initiatives include the Global Fund to Fight AIDS, Tuberculosis and Malaria; US President’s Emergency Plan for AIDS Relief; US President’s Malaria Initiative; and Global Polio Eradication Initiative. Together, these groups have contributed to a considerable reduction in illness and deaths from HIV, tuberculosis (*2*), malaria (*3*), and polio (*4*) in Nigeria. The Global Polio Eradication Initiative supported the establishment of a laboratory network, emergency operations center (EOC), 2 molecular laboratories, and enhanced vaccination efforts and provided substantial operational support for Nigeria’s polio response (*5*). Similarly, the US President’s Emergency Plan for AIDS Relief program has been the main funder of HIV-related activities in Nigeria, supporting the establishment of testing sites and laboratories, providing treatment to persons living with HIV, and accounting for 67% of the $532.4 million reported HIV spending in 2018 (*6*). By focusing resources, priorities, and policies on a single disease, these programs have achieved notable public health improvements for persons in Nigeria. A steady decline in HIV and malaria prevalence across the country has been observed, more persons are presently accessing disease testing and treatments compared with 2001 (*3*), and, in June 2020, Nigeria achieved wild polio virus–free status (*5*). However, Nigeria has experienced mixed success in using the capacities built through these donor-funded vertical programs to respond to new health threats, such as regional Ebola outbreaks and the global COVID-19 pandemic.

Although most global health initiatives are mainly focused on a single disease (*7*), program directions are largely driven by the respective donors. In some instances, these programs have created parallel systems for their respective disease(s). For example, separate sample transportation systems have been created for HIV and polio in parallel with other endemic diseases systems in Nigeria. Minimal intentional convergence of resources has been provided for these specific disease programs to strengthen the entire health system. Spillover effects on other programs have been marginal because many of the vertical programs have been implemented outside of the mainstream public health preparedness and response architecture in Nigeria.

Examples of spillover effects exist that might be instructive. During the 2014–2016 Ebola virus outbreak in West Africa, resources and experiences from the polio program in Nigeria were leveraged for Ebola response activities (*8*). The polio effort in Nigeria was well recognized worldwide (*9*), and the polio EOC was used as a coordination structure for the national response to the Ebola outbreak. In the years after that Ebola outbreak, however, polio resources were not leveraged further for other disease outbreaks and were refocused completely on the polio eradication program. Despite the prevalence of infectious diseases and annual outbreaks, Nigeria did not have a public health EOC 2 years after the Ebola response (*10*). In 2016, an integrated disease prevention and response mechanism was established through the evolution of the Nigeria Centre for Disease Control (NCDC); however, responses to HIV, tuberculosis, malaria, and polio remained primarily vertical interventions and outside of NCDC’s oversight.

When the COVID-19 pandemic began in 2020, NCDC negotiated individually with the different vertical disease programs for resources to support the response. GeneXpert systems (Cepheid, https://www.cepheid.com) originally purchased for tuberculosis testing were repurposed for SARS-CoV-2 testing, thereby contributing to the rapid expansion of the country’s testing capacities (*11*,*12*), including increased near-patient testing and turnaround time for COVID-19 case confirmation. Specifically, GeneXpert tests provided results within a 2-hour turnaround time, compared with 6 hours for reverse transcription PCR testing. Similarly, a major HIV testing laboratory, established within NCDC’s National Reference Laboratory with support from the US Centers for Disease Control and Prevention, was leveraged for high throughput testing for SARS-CoV-2, which increased testing capacity at this critical time. In Nigeria and across several countries, field epidemiologists from field epidemiology training programs were deployed to enhance the available workforce in response to the COVID-19 pandemic (*13*).

Efforts to leverage HIV and tuberculosis resources for the COVID-19 pandemic response were, however, not without challenges. For example, the tuberculosis program’s procurement and distribution of cartridges, reagents, and supplies were largely dependent on support by external partners. Therefore, integrating GeneXpert testing supplies into the national unified supply chain for the COVID-19 response proved to be difficult. Faced with global supply shortages and increased demand, the government of Nigeria had to develop a strategy to manage the shortage of supplies in government-run laboratories and a separate strategy for other laboratories that were heavily donor-dependent. In addition, the reporting systems for HIV and tuberculosis laboratories were isolated from the national surveillance system, which required harmonization of reporting tools and reporting frequency across laboratories and additional training for laboratory staff. These challenges affected the completeness and timeliness of the epidemiologic analyses.

The experiences in Nigeria demonstrated that limited integration of donor-funded vertical programs with government systems jeopardizes the sustainability of these programs and complicates the use of program resources to support emergency responses to outbreaks. However, close partnerships with government agencies and good field collaboration improved the overall response. The effectiveness of global health initiatives will very likely be improved through better coordination between donor-supported programs and government-led systems and institutions for establishing initiative priorities, design, implementation, and evaluation. Specifically, investments through global health initiatives should be reviewed in the context of government-led systems and institutions. Individual initiatives should align with approaches for other endemic diseases, even if those diseases are not priorities of donor partners. Such an approach has the potential to provide an even higher level of return on investment for donors.

Nigeria’s Presidential Task Force for COVID-19 provides an example of a government-led structure supported by donors during an emergency (*12*). The growth and increasing capacity of the National Public Health Institutes in Africa supported by the Africa Centers for Disease Control (*14*) provide an opportunity for improved convergence and coordination.

Investments in global health programs should be leveraged to improve preparedness for future pandemics. Several reports have shown that countries with higher investments in health security were better prepared to respond to the COVID-19 pandemic (*15*). Previous investments in preparedness coordinated by NCDC, such as the establishment of a public health EOC network and digitalization of the country’s surveillance system, provided a foundation for Nigeria’s COVID-19 response. Subsequent funding for HIV, tuberculosis, malaria, and polio programs should enable appropriate responses to future pandemics. Investments could potentially include the development of common standards that increase flexibility to use these funds in response to large outbreaks and pandemics, while ensuring continuity of program specific goals.

Our experience during the COVID-19 pandemic showed that pooling and unified governance of resources from various donors reduced fragmentation and increased the collective response to the pandemic. Initiatives such as the United Nations Basket Fund (*16*) and the private sector task force Coalition Against COVID-19 (*11*) enabled government leadership to direct resources toward interventions that maximized pandemic responses while providing donors with opportunities to contribute their diverse expertise and maintain financial oversight. Using such approaches in future global health interventions, especially in large countries, could reduce the risk for fragmentation.

In conclusion, strong collaborations among partners that have governments at their core will prevent or mitigate the effects of the next pandemic. The World Health Organization Hub for Pandemic and Epidemic Intelligence (*17*) was established in response to this urgent collaborative need. For example, the Hub for Pandemic and Epidemic Intelligence has begun to develop a set of principles to support data sharing across countries and disciplines. Developing and sustaining a global health security architecture enshrined in the principles of mutual trust and equity for all is not only necessary but is a critical approach to mitigate the next pandemic.
